# Catheter‐Based Therapies for Intermediate‐High Risk Pulmonary Embolism: A Protocol for a Systematic Review With Bayesian Pairwise and Network Meta‐Analyses

**DOI:** 10.1111/aas.70306

**Published:** 2026-07-17

**Authors:** Jacob Hindborg Hovmann, Thomas Skjoldborg Sornn‐Friese, Frederik Færgemann Lau, Niels Brandt, Isak Mazanti Cold, Janus C. Jakobsen, Jasmin Dam Lukoschewitz, Alasdair D. Henderson, Anne Sophie Overgaard Olesen, Jens Dahlgaard Hove, Jesper Kjærgaard, Johannes Grand

**Affiliations:** ^1^ Department of Cardiology Copenhagen University Hospital–Amager‐Hvidovre Hospital Copenhagen Denmark; ^2^ Department of Cardiology Copenhagen University Hospital–Rigshospitalet Copenhagen Denmark; ^3^ Copenhagen Trial Unit, Centre for Clinical Intervention Research, Capital Region of Denmark Rigshospitalet, Copenhagen University Hospital Copenhagen Denmark; ^4^ Department of Regional Health Research, The Faculty of Health Sciences University of Southern Denmark Odense Denmark; ^5^ School of Cardiovascular and Metabolic Health University of Glasgow Glasgow UK; ^6^ Department of Cardiology Copenhagen University Hospital – Bispebjerg‐Frederiksberg Copenhagen Denmark; ^7^ Department of Clinical Medicine University of Copenhagen Copenhagen Denmark

**Keywords:** Bayesian pairwise meta‐analyses, catheter‐directed therapy, intermediate‐high risk, network meta‐analysis, pulmonary embolism, systematic review, thrombectomy, thrombolysis

## Abstract

**Background:**

Intermediate‐high risk pulmonary embolism (PE) represents a clinically challenging subgroup of acute PE, characterized by right ventricular dysfunction and increased risk of hemodynamic deterioration despite treatment with anticoagulation therapy. Although systemic thrombolysis may provide more rapid reperfusion, its use is limited by an increased risk of major bleeding. Catheter‐based treatment strategies have emerged as potential alternatives, but their comparative efficacy and safety remain uncertain.

**Objective:**

To assess the benefits and harms of catheter‐based treatment strategies compared with contemporary control regimens, including standard anticoagulation with or without systemic thrombolysis, in patients with intermediate‐high risk PE.

**Methods:**

This protocol outlines a systematic review with Bayesian pairwise meta‐analysis and network meta‐analysis of randomized clinical trials evaluating catheter‐based therapies for hospitalized patients with intermediate‐high risk PE. Eligible interventions include catheter‐directed thrombolysis, ultrasound‐assisted thrombolysis, and catheter‐based embolectomy. Comparators may include standard anticoagulation alone or in combination with systemic thrombolysis or other active control regimens reflecting standard of care. Searches will be conducted in relevant databases, supplemented by trial registries. Two reviewers will independently screen studies, extract data, and assess risk of bias using the Cochrane Risk of Bias 2 tool. Bayesian pairwise meta‐analyses and network meta‐analysis will be performed using weakly informative priors to synthesize direct and indirect evidence across interventions. Results will be reported as posterior effect estimates with 95% credible intervals. The primary outcome will be all‐cause mortality. Secondary and exploratory outcomes include major bleeding as defined according to the criteria of the International Society on Thrombosis and Haemostasis within 7 days, hospital length of stay, and change in RV/LV ratio within 48 h. GRADE assessments will be performed where applicable.

**Discussion:**

This review will synthesize direct and indirect randomized evidence on catheter‐based treatment strategies for intermediate‐high risk PE. The findings may help clarify the role of these interventions in contemporary management, inform future guideline recommendations, and identify persistent evidence gaps requiring further large‐scale randomized trials.

## 1 Introduction

Pulmonary embolism (PE) is a cardiovascular emergency that can lead to acute hemodynamic compromise, interfering with both circulation and gas exchange [1]. PE remains a significant cause of cardiovascular morbidity and mortality worldwide, as the third most common cause of cardiovascular death, after myocardial infarction and stroke [2]. It is estimated that the annual incidence ranges from 39 to 115 per 100,000 population [3].

PE occurs when a thrombus, typically originating in the deep venous system, dislodges and travels through the venous circulation to the pulmonary arteries, causing partial or complete vascular obstruction. PE‐induced vasoconstriction contributes to the early increase in pulmonary vascular resistance (PVR) following embolic obstruction [4, 5]. The abrupt elevation in PVR imposes an acute pressure overload on the right ventricle (RV), resulting in RV dilation and altering the contractile function of the RV myocardium via the Frank–Starling mechanism [6, 7]. This impairs LV filling, reduces cardiac output, and contributes to systemic hypotension and hemodynamic compromise [8].

According to the European Society of Cardiology (ESC) guidelines, patients with PE are stratified into four risk categories: low risk, intermediate‐low risk, intermediate‐high risk, and high risk [6]. Based on guidelines, patients with low‐ and intermediate‐low risk PE can, in most cases, be managed with oral anticoagulation. In patients with high‐risk PE, defined by persistent hypotension or shock, systemic thrombolysis in addition to anticoagulation with heparin should be considered first‐line therapy [6].

Between these two risk categories lies the group of patients with intermediate‐high risk PE, accounting for approximately 10% of total cases [9]. According to ESC guidelines, patients in this group are hemodynamically stable, as well as presenting a PESI Class III–IV, distinguishing them from low‐risk PE. Differentiating intermediate‐high/low‐risk relies on a display of RV dysfunction and elevated cardiac biomarker levels. Although these patients are often treated with anticoagulation, they remain at increased risk of clinical deterioration [6], and the optimal treatment of this subgroup remains a matter of ongoing debate. Due to the increased risk of major bleeding when treating patients with systemic thrombolysis compared with stand‐alone anticoagulation, catheter‐based treatment has emerged as an attractive alternative in recent years [10].

**FIGURE 1:**
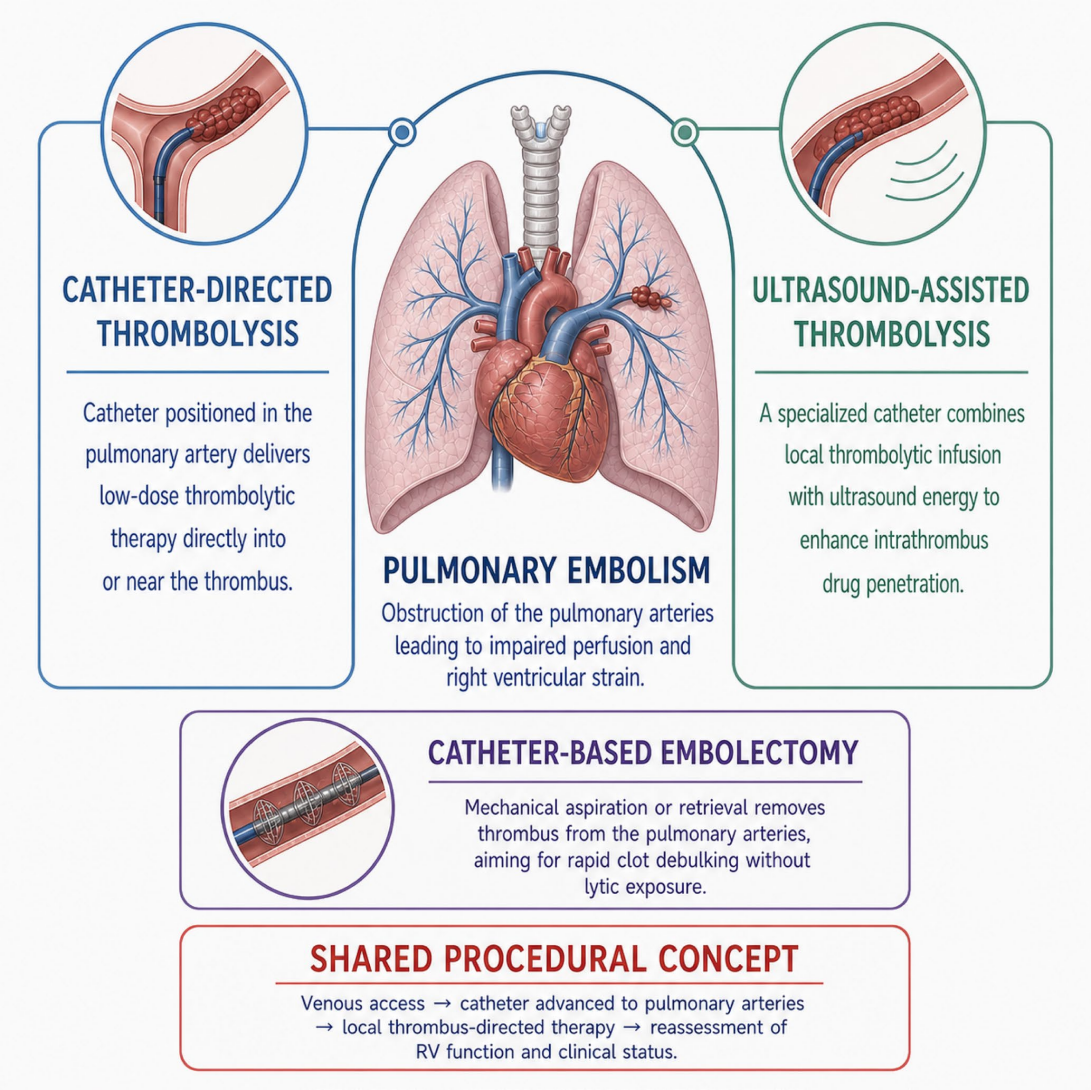
The three principles of catheter‐based therapies. Biorender and AI were used for the development of this figure.

Catheter‐directed thrombolysis (CDT) delivers a thrombolytic agent directly into the PE via a catheter advanced from the femoral or jugular vein [11]. Building on this technique, ultrasound‐assisted thrombolysis (USAT) uses a specialized catheter that both infuses thrombolytic therapy and emits ultrasound energy, with the aim of enhancing intrathrombus drug penetration and promoting fibrin disaggregation [12, 13]. This technique has been shown to improve the RV/LV ratio with low rates of major bleeding in multiple studies [14, 15, 16], but has not been shown to be superior to low‐dose systemic thrombolysis [17]. In patients with contraindications to thrombolysis or in whom catheter‐directed lytic strategies fail or are deemed insufficient, catheter‐based embolectomy and surgical pulmonary embolectomy constitute alternative reperfusion modalities [6]. An overview of the three catheter‐based treatment principles is provided in Figure 1.

### 1.1 Why Is This Review Important

Despite advances in the management of PE, the optimal treatment strategy for patients with intermediate‐high risk PE remains uncertain. Multiple reviews and meta‐analyses have evaluated this field recently; however, they all consist mainly of observational studies or a few randomized controlled trials (RCTs) [18, 19, 20]. Several RCTs have since concluded with new emerging results, including STRATIFY, HI‐PEITHO, and PEERLESS, thereby expanding the evidence base for catheter‐directed and mechanical reperfusion therapies in PE [17, 21, 22]. A systematic review and network meta‐analysis is therefore warranted to synthesize the current evidence and help clarify the optimal treatment strategy for this patient population.

### 1.2 Primary Review Question

Among patients with intermediate‐high risk acute PE, what are the effects of catheter‐based treatment strategies—CDT, USAT, or catheter‐based embolectomy—compared with contemporary control regimens such as standard anticoagulation with or without systemic thrombolysis on clinical outcomes?

### 1.3 Secondary Review Question

Among patients with intermediate‐high risk acute PE, how do catheter‐based treatment strategies—CDT, USAT, or catheter‐based embolectomy—compare with each other in terms of efficacy and safety?

## 2 Methods

This protocol is conducted in accordance with the Preferred Reporting Item for Systematic Reviews and Meta‐Analysis Protocols (PRISMA‐P) statement [23] and is registered in the PROSPERO database (under registration).

### 2.1 Criteria for Considering Studies for This Review

#### 2.1.1 Types of Trials

We will include RCTs regardless of publication status, year, and language. Quasi‐randomized and cluster‐randomized trials will not be eligible.

#### 2.1.2 Types of Participants

Participants include all patients hospitalized with intermediate‐high risk PE (as defined by trialists).

#### 2.1.3 Types of Interventions

We will include interventions consisting of CDT, USAT, or catheter‐based embolectomy for the treatment of intermediate‐high risk PE.

#### 2.1.4 Types of Control

Trials will be included if the intervention is compared to other active interventions, to placebo, or to contemporary “standard of care,” for example, heparin with or without low‐dose or high‐dose systemic thrombolysis.

#### 2.1.5 Types of Outcomes

We will include studies regardless of their respective outcomes.

### 2.2 Use of Artificial Intelligence (AI) Tools

AI assistance (ChatGPT) was used for the development of a graphical figure and for minor linguistic refinement of selected sentences to improve clarity and readability.

### 2.3 Outcome Measures

#### 2.3.1 Primary Outcomes

The primary outcome will be all‐cause mortality, assessed at the longest available follow‐up in each trial.

#### 2.3.2 Secondary Outcomes

The secondary outcomes will be (1) major bleeding as defined according to the criteria of the International Society on Thrombosis and Haemostasis within 7 days, (2) hospital length of stay, (3) clinical deterioration including escalation to bailout therapy and/or ICU admission, (4) readmission at 30 days, and (5) serious adverse events.

#### 2.3.3 Exploratory Outcomes

The exploratory outcomes will be (1) change in RV/LV ratio within 48 h, (2) reduction in refined Miller score at 48–96 h, (3) dyspnea scores (mMRC/NYHA) at 1–3 months, (4) pulmonary artery pressure, (5) 6‐min walk test at 3‐month follow‐up, and (6) vital parameters including blood pressure, heart rate, and oxygen supplementation.

### 2.4 Search Methods

We will search Medline, Embase, Cochrane Central Register of Controlled Trials, Latin American and the Caribbean Literature on Health Sciences, and Web of Science. The search will begin in May 2026.

#### 2.4.1 Searching Other Resources

Reference lists of relevant trials will be checked for unidentified randomized trials. Searches for ongoing or recent trials will be conducted in:

- ClinicalTrials.gov (http://www.clinicaltrials.gov)
- Google Scholar (https://scholar.google.dk/)
- European Medicines Agency (EMA) (https://www.ema.europa.eu/ema/)
- US Food and Drug Administration (FDA) (http://www.fda.gov)
- National Medical Products Administration (NMPA) (https://english.nmpa.gov.cn/)
- Medicines and Healthcare products Regulatory Agency (https://www.gov.uk/government/organisations/medicines‐and‐healthcare‐products‐regulatory‐agency)
- The World Health Organization (WHO) International Clinical trials Registry Platform (ICTRP) search portal (http://apps.who.int/trialsearch)
- Clinical Trials Registry Platform (http://www.who.int/ictrp/en/).

### 2.5 Selection Process

Two authors (J.H.H. and T.S.S.‐F.) will independently screen all relevant articles by title, abstract, and full text using the Covidence systematic review software (Veritas Health Innovation; available at www.covidence.org). The software will aim to remove all duplicates. After initial screening, all conflicts will be resolved through discussion or by a third reviewer (J.G.). Articles passing the initial screening will be reviewed in full text, independently by the same two authors. We will include a PRISMA flow diagram showing the number of studies remaining after each stage of the selection process with reasons for exclusion.

#### 2.5.1 Data Extraction and Management

Two authors (J.H.H. and T.S.S.‐F.) will independently extract data from eligible trials. Discrepancies will be resolved through discussion or with a third author (J.G.). If additional data is needed, authors will be contacted by email.

### 2.6 Trial Characteristics

The following data will be extracted as relevant:

- General information
  ○ First author name
  ○ Year of publication
  ○ Geographical location of the study (country)
  ○ Study design
  ○ Inclusion and exclusion criteria
  ○ Time period of patient enrolment
  ○ Number of patients randomized and analyzed (sample size)
  ○ Intervention and control groups
  ○ Length of follow‐up
- Participants
  ○ Summary demographics
    - Age (mean/median)
    - Sex (proportion of females)
    - Comorbidities
  ○ PE risk stratification: intermediate‐high risk definition (e.g., PESI III–IV, RV dysfunction and elevated cardiac biomarkers)
  ○ Baseline RV/LV ratio
  ○ Baseline troponin/BNP levels
  ○ Baseline anticoagulation medication (anticoagulation type: heparin/DOAC/warfarin)
- Intervention details
  ○ Intervention type
  ○ Catheter type
  ○ Thrombolytic agent and dose
  ○ Duration of infusion
- Relevant results

#### 2.6.1 Risk of Bias in Individual Studies

The risk of bias assessment will be assessed independently by two reviewers using the Cochrane Risk of Bias tool version 2 (RoB2) [24]. Any disagreements will be resolved through discussion or, if needed, with the involvement of a third reviewer (J.G.).

### 2.7 Measures of Treatment Effect and Data Synthesis

We will use risk ratios (RRs) with 95% credible intervals (CrIs) for dichotomous outcomes. For continuous outcomes, we will use mean differences (MDs) with 95% CrIs when the same scale is used across trials, and standardized mean differences (SMDs) with 95% CrIs when outcomes are measured using different scales. For time‐to‐event outcomes, we will use hazard ratios (HRs) with 95% CrIs when reported.

We will provide a descriptive summary of all included trials and present relevant trial characteristics in tables. All meta‐analyses will be conducted within a Bayesian framework using weakly informative priors to assume no prior knowledge about the efficacy of catheter‐based treatments. For direct comparisons, we will perform Bayesian pairwise meta‐analyses. Given the expected clinical and methodological heterogeneity across trials, random‐effects models will be considered the primary analyses. The prior for between‐study heterogeneity will be informed by the work of Turner et al. [25]. If a small number of studies (< 5) are identified as eligible, then a more informative prior on between‐study heterogeneity will be used. Bayesian parallels of fixed‐effect models (strongly informative prior of between‐study heterogeneity almost zero) will be used as sensitivity analyses. Results will be interpreted considering the posterior effect estimates, the width of the CrIs, and the degree of between‐study heterogeneity. Between‐study heterogeneity will be assessed using posterior estimates of the between‐study heterogeneity parameter and corresponding 95% CrIs, together with visual inspection of forest plots. We will additionally prespecify a minimally clinically important difference of 2 percentage points on the absolute risk difference scale for dichotomous clinical outcomes. For efficacy outcomes, we will estimate the posterior probability that catheter‐based therapy reduces the absolute risk by ≥ 2%, whereas for safety outcomes we will estimate the posterior probability that catheter‐based therapy increases the absolute risk by ≥ 2%. These analyses will be considered supportive and interpreted alongside posterior effect estimates, 95% CrIs, heterogeneity, and certainty of evidence.

If the assumptions underlying network meta‐analysis are considered acceptable, we will perform a Bayesian network meta‐analysis using hierarchical models to synthesize direct and indirect evidence across all eligible interventions. The validity of the network meta‐analysis depends on the assumptions of transitivity and consistency. Transitivity will be assessed by evaluating whether important clinical and methodological effect modifiers are similarly distributed across comparisons. Consistency will be assessed by comparing direct and indirect evidence where both are available. Results will be reported as posterior median effect estimates with 95% CrIs. Treatment rankings will be presented if appropriate but interpreted with caution. The data analysis will be conducted using R with appropriate Bayesian meta‐analysis software packages [26].

The certainty of evidence will be assessed using GRADE.

If quantitative synthesis is inappropriate, we will summarize results narratively.

## 3 Discussion

This systematic review applies a Bayesian framework with both pairwise and network meta‐analyses to synthesize current evidence on the comparative efficacy and safety of catheter‐based therapies versus standard anticoagulation alone or in combination with systemic thrombolysis or other active control regimens reflecting standard of care in patients with intermediate‐high risk PE. In the absence of prior comprehensive network meta‐analysis incorporating multiple randomized trials, our study addresses this critical evidence gap and may inform guideline recommendations, multidisciplinary protocols, and specific treatment strategies.

The management of intermediate‐high risk PE remains clinically challenging. Anticoagulation therapy serves as the cornerstone of intermediate‐high risk PE management. However, as thrombus resolution may be slow, patients remain vulnerable to early clinical deterioration [6]. Systemic thrombolysis may provide more rapid reperfusion, but this benefit should outweigh a substantially increased risk of major bleeding and no clear mortality benefit compared to anticoagulation alone [10, 27]. Catheter‐based therapies, including CDT, USAT, and catheter‐based embolectomy, could potentially bridge this gap, with recent American clinical practice guidelines stating that CDT and catheter‐based embolectomy may be considered in selected patients [28].

Although these catheter‐based therapies may achieve local debulking of the embolism, potentially resulting in faster RV recovery and lower bleeding rates than systemic thrombolysis [29], their effect on clinical endpoints remains uncertain. Existing studies vary with respect to patient selection, treatment protocols, dosing, and outcome reporting, which makes the evidence difficult to interpret and compare across interventions. A network meta‐analysis may therefore be particularly useful, as it allows synthesis of both direct and indirect evidence across multiple treatment strategies.

Implementation of these techniques demands meticulous, long‐term planning, including establishment of a multidisciplinary infrastructure and prospective assessment through multiple RCTs to validate efficacy and safety across heterogeneous cohorts. Currently, such treatments are primarily available in highly specialized cardiac centers [29]. The introduction of catheter‐based techniques is intuitively appealing—a pattern familiar from other invasive interventions such as intra‐aortic balloon pumping [30]. This intuitive appeal often stems from the assumption that technologically sophisticated, minimally invasive approaches will translate into improved clinical outcomes. However, experience has shown that even promising preclinical or early‐phase results may fail to yield meaningful benefits when tested against robust clinical endpoints [30]. Preliminary studies suggest that catheter‐based approaches may serve as a valuable adjunct to existing therapeutic strategies, particularly in patients for whom systematic thrombolysis is contraindicated [29].

This protocol outlines a framework for catheter‐based treatment and mechanical thrombectomy of intermediate‐high risk PE, with provisions for a systematic review with Bayesian pairwise and network meta‐analysis to synthesize direct and indirect evidence across interventions. Potential limitations of this study include the small number of available RCTs and the frequent use of surrogate rather than hard clinical endpoints, which may reduce the certainty and clinical applicability of pooled estimates. Further limitations include heterogeneity in risk‐stratification definitions across RCTs, with some studies categorizing patients into intermediate‐risk subgroups and often lacking explicit delineation of the intermediate‐high risk subgroup, as well as variability in catheter‐directed therapy protocols, both of which may introduce clinical heterogeneity and limit the strength of indirect comparisons in the network meta‐analysis.

## Author Contributions

**Jacob Hindborg Hovmann:** writing – original draft, project administration, methodology, investigation, data curation, conceptualization. **Thomas Skjoldborg Sornn‐Friese:** writing – review and editing, project administration, methodology, investigation, data curation, conceptualization. **Frederik Færgemann Lau:** writing – review and editing, validation. **Niels Brandt:** writing – review and editing, validation. **Isak Mazanti Cold:** writing – review and editing, validation. **Janus C. Jakobsen:** writing – review and editing, validation. **Jasmin Dam Lukoschewitz:** writing – review and editing, validation. **Alasdair D. Henderson:** writing – review and editing, data curation, validation. **Anne Sophie Overgaard Olesen:** writing – review and editing, data curation, validation. **Jens Dahlgaard Hove:** writing – review and editing, validation. **Jesper Kjærgaard:** writing – review and editing, validation. **Johannes Grand:** writing – review and editing, supervision, project administration, methodology, conceptualization.

## Funding

The authors have nothing to report.

## Conflicts of Interest

The authors declare no conflicts of interest.

## Data Availability

The data that support the findings of this study are available from the corresponding author upon reasonable request.
